# A case of Meigs syndrome mimicking metastatic breast carcinoma

**DOI:** 10.1186/1477-7819-7-10

**Published:** 2009-01-22

**Authors:** Sophocles Lanitis, Sivahamy Sivakumar, Kasim Behranwala, Emmanouil Zacharakis, Ragheed Al Mufti, Dimitri J Hadjiminas

**Affiliations:** 1 General Surgery Department, St Mary's Hospital, Imperial College Healthcare NHS Trust, Praed Street, London, W2 1NY, UK; 2 Department of Biosurgery and Surgical Technology, Imperial College London 10th Floor, QEQM Wing, St. Mary's Campus, Praed Street, London, W2 1NY, UK

## Abstract

**Background:**

Adnexal masses are not uncommon in patients with breast cancer. Breast cancer and ovarian malignancies are known to be associated. In patients with breast cancer and co-existing pleural effusions, ascites and adnexal masses, the probability of disseminated disease is high. Nevertheless, benign ovarian masses can mimic this clinical picture when they are associated with Meigs' syndrome making the work-up and management of these patients challenging. To our knowledge, there are no similar reports in the literature and therefore we present this case to highlight this entity.

**Case presentation:**

A 56-year old woman presented with a 4 cm, grade 2, invasive ductal carcinoma of her left breast. Pre-treatment staging investigations showed a 13.5 cm mass in her left ovary, a small amount of ascites and a large right pleural effusion. Serum tumour markers showed a raised CA125 supporting the malignant nature of the ovarian mass. The cytology from the pleural effusion was indeterminate but thoracoscopic biopsy failed to show malignancy. The patient was strongly against mastectomy and she was commenced on neo-adjuvant Letrozole 2.5 mg daily with a view to perform breast conserving surgery. After a good response to the hormone manipulation, the patient had breast conserving surgery, axillary sampling and laparoscopic excision of the ovarian mass which was eventually found to be a benign ovarian fibroma.

**Conclusion:**

Despite the high probability of disseminated malignancy when an ovarian mass associated with ascites if found in a patient with a breast cancer and pleural effusion, clinicians should be aware about rare benign syndromes, like Meigs', which may mimic a similar picture and mislead the diagnosis and management plan.

## Background

With the increased incidence of breast cancer, along with the concurrent advances of the imaging modalities is not uncommon to find adnexal masses during the preoperative work-up of these patients [1].

Breast cancer is associated with either primary or secondary ovarian cancer since the risk for ovarian malignancies is twofold among breast cancer patients [1,2].

Moreover, among breast cancer patients, ovarian cancer is the most common second malignancy found [1,3] and this association, makes determination of the nature of these ovarian masses challenging whilst managing a case of breast cancer [1].

An enlarged adnexal mass in a breast cancer patient over 50-years old, especially when associated with ascites and pleural effusion favors the diagnosis of malignant involvement and should be extensively investigated and managed accordingly [1,2,4,5]. Nevertheless, there are occasions that a benign condition can present with such a dramatic picture [5]. The presence of a benign ovarian mass, associated with ascites and pleural effusion that resolve after the resection of the adnexal mass define Meigs' syndrome [5-10].

In breast cancer patients, benign ovarian masses associated with Meigs' syndrome can mimic the clinical picture of extensive carcinomatosis making the work-up and management of these patients challenging. To our knowledge, there are no similar reports in the literature and therefore we present this case in order to highlight this entity.

## Case presentation

A 56-year-old Caucasian woman presented with a 4 cm in diameter lump in her left breast. She had a screening mammogram done 3 years earlier which was reported as suspicious but the patient did not seek medical attention for this period. She was otherwise fit and well without any significant past medical history. She was not on any medications and did not have previous admissions to a hospital. She did not have any family history of any form of cancer.

The patient underwent a triple assessment for the breast lump which was found to be suspicious in both the clinical and imaging investigations.

The mass was confirmed to be a grade II invasive ductal carcinoma on core biopsy which was strongly positive for estrogen (ER) receptors while it was negative for progesterone (PgR) receptors. The tumor was HER-2 negative.

During pre-treatment, staging investigations, which included computerized tomography (CT) scan of the chest and abdomen, she was found to have a 13.5 cm mass in her left ovary, a small amount of ascites and a large right pleural effusion. The pelvic ultrasound showed a 13.5 cm × 10 cm × 8 cm hypo-echoic ovarian mass with an irregular necrotic, also hypo-echoic central area and moderate amount of ascitis.

**Figure 1:**
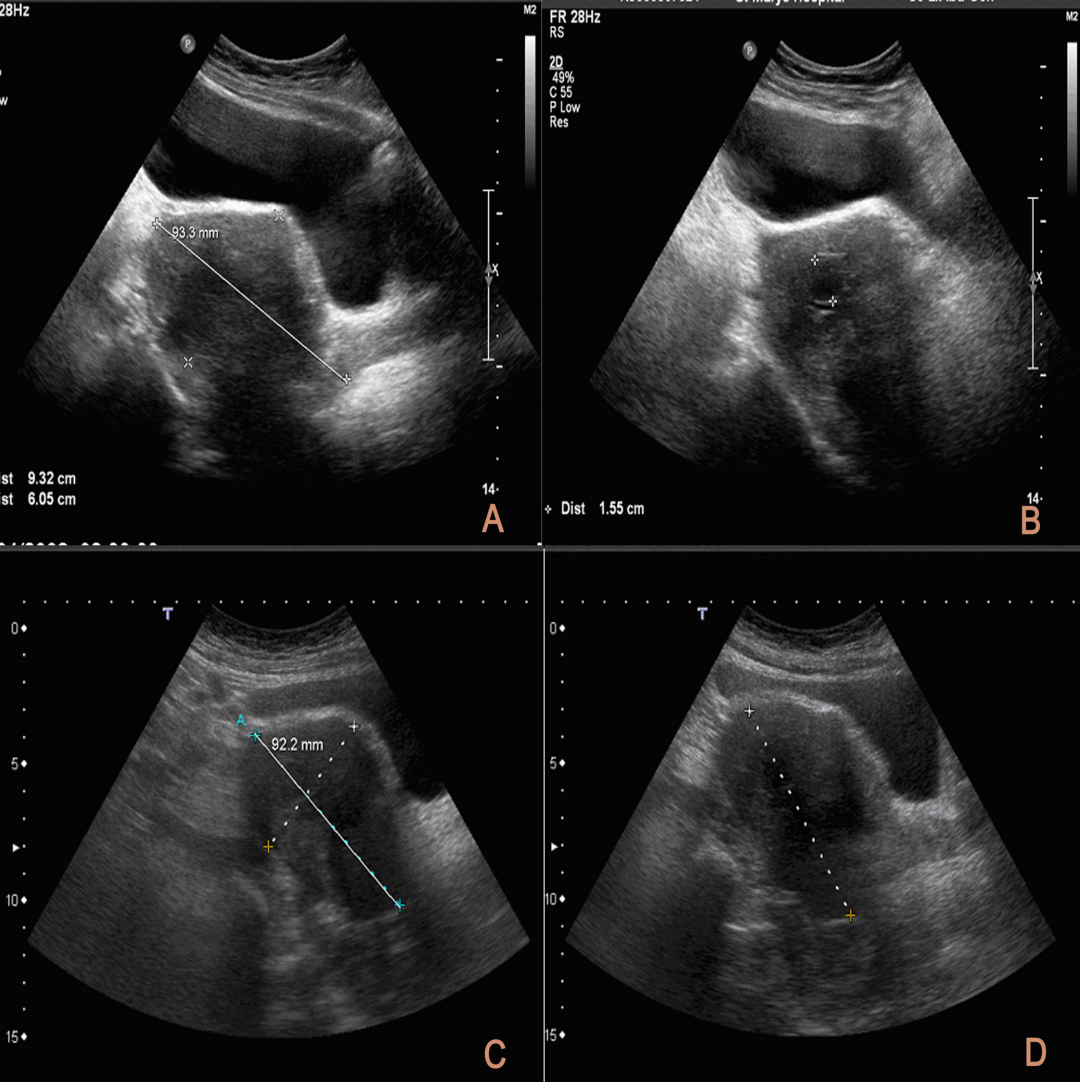
**Ultrasound (U/S) of the pelvis**. (A) 1 year after the diagnosis showing a reduced size (93.3 mm) hypo-echoic ovarian mass and resolution of the ascites. (B) 2 years after the diagnosis showing the unchanged ovarian mass and an irregular necrotic area in the centre (also present on previous scans). (C/D) 3 years after the diagnosis showing no progression and rather an improvement of the disease.

Considering the common presentation of ovarian carcinomas with similar picture and the association of breast cancer with ovarian carcinomas, initially the ovarian mass was thought to be metastatic as was the pleural effusion. Serum tumor markers showed a raised CA125, (59 u/ml with normal values < 24) supporting the malignant nature of the ovarian mass. The pleural effusion was aspirated but cytology was indeterminate. Aspiration of the pleural effusion caused a pneumothorax. Due to persistent fluid drainage through the chest tube, the patient eventually underwent thoracoscopic pleurodesis with simultaneous biopsy of the pleura, 6 months after diagnosis. The pleural effusion did not recur after this procedure and the pleural biopsy taken at the time showed no malignancy. The patient from the beginning was strongly against mastectomy and she was commenced on neo-adjuvant Letrozole 2.5 mg daily with a view to perform breast conserving surgery later. The breast cancer became impalpable within 1 year and continued to respond to Letrozole. Meanwhile, regularly repeated pelvic ultrasounds initially showed a reduction of the ovarian mass size (Fig 1A), which had an irregular necrotic area in its centre (Fig 1B), and then an unchanged picture (Fig. 1C and 1D) without any progression of the disease. Repeated CA 125 values showed a decline and subsequently a normalization of the value (15 u/ml) during the following 3 years. All these changed our initial impression about the malignant nature of the ovarian mass and the extent of the breast cancer. Since, the breast cancer size plateau at 1 cm and 3 years after the diagnosis the patient was advised and persuaded to have some surgery. She only agreed to have wire – guided excision of the breast primary lesion, sentinel node biopsy and axillary sampling. Despite the indication for hysterectomy and bilateral salpingo-oophorectomy, the patient declined extensive procedures and agreed only to have the ovarian mass excised laparoscopically. During the laparoscopy there was no residual ascitis, the ovarian tumor was mobilized laparoscopically and removed through a small Pfannestiel incision extending horizontally to the left of the midline only.

**Figure 2:**
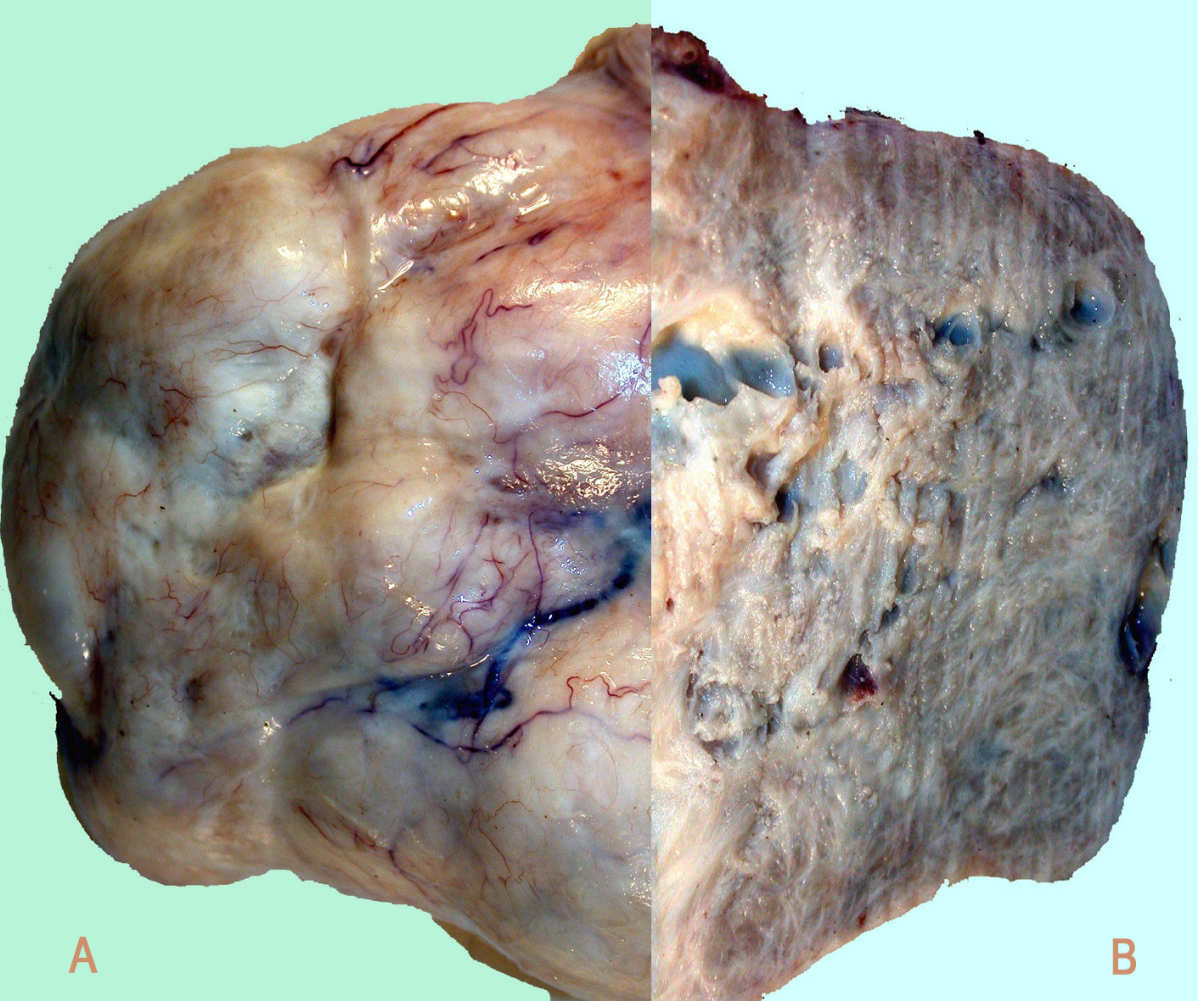
**Macroscopic pictures of the ovarian mass specimen**. (A) Uncut (firm, solid mass). (B) Cross-section of the specimen.

Histological examination of the 11 cm firm, solid ovarian mass (Fig. 2) confirmed the presence of a benign ovarian fibroma. Her breast cancer was completely excised with good margins but the sentinel lymph node contained metastasis while 2 of 4 sampled nodes contained isolated tumor cells on immunohistochemistry. Since the patient declined axillary clearance, she was referred for post-operative radiotherapy to the breast and axilla. The CA 125 remained within the normal range postoperatively (15 u/ml).

## Discussion

Apart from the known association of primary ovarian cancer with breast cancer in BRCA mutation carriers, breast secondary ovarian deposits are also common. This has been demonstrated in series of breast cancer patients undergoing oophorectomy either for diagnostic or adjuvant purposes. If there is no selection of the patients according to the preoperative suspicion of metastatic disease, 26%–50% of these will have malignancy mostly metastatic from the breast (30%–50%) [2,11].

Moreover, palliative oophorectomy for metastatic breast can reveal up to 20% incidence of metastatic disease in the ovaries [2]. It has been reported that in up to 30% of staging laparoscopies secondary ovarian deposits are commonly of breast origin [2].

In women over 50 years, more than 40% of ovarian neoplasms will be malignant [2]. The risk of malignancy when an ovarian mass is found increases with the stage of the breast cancer while other risk factors include the enlarged size of the adnexal mass over 5 cm, the complexity of the mass shown by the ultrasound and the raised cancer antigen (CA) CA-125 [1].

On the other hand younger patients without ascites or any other signs of disseminated disease will mostly have a benign histology in up to 78% [1].

Meigs' syndrome represents a benign condition which can present with a dramatic picture [5] since the syndrome is defined by the presence of a benign ovarian mass, associated with ascites and pleural effusion that resolve after the resection of the adnexal mass [5-10].

Despite earlier similar reports, Meigs' properly described the triad of the syndrome, initially in his book "Tumours of the female Pelvic organs". Subsequently he published along with Cass a series of 7 patients with fibromas of the ovaries and the associated syndrome in 1937 [6]. Fibromas account for 4% of ovarian neoplasms and along with fibrothecomas are the most common benign ovarian mass associated with the syndrome (91.4%) [5,9,10,12]. These tumours have an extremely low malignant potential and they present during the fifth and sixth decade of the life [5]. Ten to 15% of all fibromas are associated with ascites while only 1% have pleural effusion in addition to ascites [4,10]. On ultrasound, ovarian fibromas typically appear as homogeneous solid hypoechoic masses with strong posterior acoustic attenuation, though larger masses frequently present with more heterogeneous components. In these cases hyperechoic areas represent calcification and more hypoechoic segments representing cystic degeneration[13,14].

Apart from the aforementioned benign tumours, Brenner tumours and granulosa cell tumours can be associated with the syndrome in a smaller percentage of the cases [4,9,10].

Other benign or malignant pelvic tumours associated with ascites and pleural effusion are described as pseudo-Meigs' syndrome [4,9]

Any breast cancer patient found to have ascites, pleural effusion and adnexal mass should be investigated thoroughly for possible malignancy bearing though in mind that benign conditions like Meigs' syndrome may present with a similar picture [4,5].

The work-up should include ultrasound (US) of the pelvis, CT of the chest abdomen and pelvis, magnetic resonance imaging (MRI) of the pelvis, sampling of the pleural as well as the ascitic fluid, and serum markers of malignancy like CA125 [4,5]. The pleural and peritoneal fluid should be assessed to determine whether their composition is consistent with an exudate or a transudate [5]. In Meigs' syndrome, the pleural effusion is usually unilateral (75%) with a predominance of the right side (65%) [5,12]. Moreover, the fluid can be sent for cytology which may confirm malignancy [4,9,10]. The pleural fluid in Meigs' syndrome has the same characteristics as that of the ascites and it is believed to be caused from the lymphatic flow across the diaphragm through the transdiaphragmatic system [5,9,12,15].

Cases with Meigs' syndrome and elevated CA125, which is indicative of epithelial ovarian cancer, have been reported [4,10]. CA125 is raised in 80% of patients with advanced ovarian cancer and despite the fact that it cannot be used for screening purposes it is useful in assessing the response to treatment as well as for detecting recurrences during follow up [10]. In patients with benign pelvic tumours, a significantly raised CA125 can be found in up to 11.5% while mild to moderate raise of the marker can be found in up to 22% of such patients especially those with associated ascites [4,10,16]. A positive for malignancy fine needle aspiration cytology (FNA) of the ascitic fluid in patients with raised CA125 can only be false positive in 0.3% of the cases [4,10].

The ascitic fluid collection related to benign ovarian tumours is thought to be caused by excessive transudate from the tumours surface in a degree that the peritoneum cannot absorb [4]. There are various theories about the pathophysiology of pleural effusion of which one supports the quick transfer of the ascitic fluid via transdiaphragmatic lymphatic channels or stomas [17]. The rapid transfer was demonstrated using dyes and radiolabelled albumin which were injected into the lower abdomen in patients with Meigs' syndrome and detection of the tracers in the right pleura within 3 hours [18].

The prognosis of Meigs' syndrome is extremely good, and resection of the involved ovary leads to complete resolution of the pleural and peritoneal fluid with no further recurrence while otherwise the fluid is persistent [5,7,8,10]. In our case the thoracoscopic pleurodesis used to control the persistent drainage from the chest drain eventually controlled the pleural effusion. Moreover, despite the lack of similar evidence in the literature, Letrozole used as neoadjuvant for the breast cancer reduced by 2 cm the size of the ovarian fibroma and the amount of the ascitic fluid to minimum. There was no evidence histologically that the ovarian mass was anything other than a fibroma and certainly there was no residual metastatic breast carcinoma on histology. The breast tumor showed a good but by no means complete response to letrozole treatment and therefore it would be difficult to believe that an ovarian metastasis would have responded completely.

Considering the good prognosis of Meigs' syndrome, prompt and accurate diagnosis to differentiate the syndrome from disseminated carcinomatosis is advisable.

## Conclusion

Despite the high probability of disseminated malignancy when an ovarian mass associated with ascites if found in a patient with a breast cancer and pleural effusion clinicians should be aware about rare benign syndromes, like Meigs', which may mimic similar picture and mislead the diagnosis and management.

## Competing interests

The authors declare that they have no competing interests.

## Authors' contributions

SL and KB collected the data, and reviewed the literature. SS tracked, reviewed and summarized the case notes and follow-up appointments. SL wrote the paper with the assistance of KB and SS. RAM and EZ reviewed and edited the initial manuscript to its final form. DJH performed the initial operation, and organized the primary management plan of the patient. He supervised the writing and editing of the paper. All authors read and approved the final manuscript.

## Consent

Written informed consent was obtained from the patient for publication of this case report and any accompanying images. A copy of the written consent is available for review by the Editor-in-Chief of this journal.
